# The angular nature of road networks

**DOI:** 10.1038/s41598-017-04477-z

**Published:** 2017-06-27

**Authors:** Carlos Molinero, Roberto Murcio, Elsa Arcaute

**Affiliations:** 1 Centre for Advanced Spatial Analysis (CASA), UCL, 90 Tottenham Court Rd., London, W1T 4TJ UK; 2 Consumer Data Research Centre (CDRC), UCL, Pearson Building, Gower Street, London, WC1E 6BT UK

## Abstract

Road networks are characterised by several structural and geometrical properties. The topological structure determines partially the hierarchical arrangement of roads, but since these are networks that are spatially constrained, geometrical properties play a fundamental role in determining the network’s behaviour, characterising the influence of each of the street segments on the system. In this work, we apply percolation theory to the UK’s road network using the relative angle between street segments as the occupation probability. The appearance of the spanning cluster is marked by a phase transition, indicating that the system behaves in a critical way. Computing Shannon’s entropy of the cluster sizes, different stages of the percolation process can be discerned, and these indicate that roads integrate to the giant cluster in a hierarchical manner. This is used to construct a hierarchical index that serves to classify roads in terms of their importance. The obtained classification is in very good correspondence with the official designations of roads. This methodology hence provides a framework to consistently extract the main skeleton of an urban system and to further classify each road in terms of its hierarchical importance within the system.

## Introduction

The search for a science of urban processes has generated growing interest from many different perspectives^1–5^. One field that has particularly attracted attention, is the study of road networks, one of the most prototypical and studied network types^6, 7^. Road networks condense in its intricate configuration a countless number of interventions which result from a myriad of historical and political micro-decisions. These have paved the way to a hierarchical structure that can be revealed if the system is studied as a percolating process^8^.

Many physical processes occurring in nature can be explained as a percolating phenomenon^9^. As a consequence, percolation theory has found a wide range of applications: e.g. for oil extraction^10^; for the study of the electrical conductivity of materials^11^, of polymerization processes^12^, of fire spreading^13^, of epidemiology^14^, and of other health aspects such as obesity^15^; and to understand the modular and integrated structure of brain networks^16^. In more technical terms, percolation theory aims to study how geometrical microscopical properties affect the macroscopic configuration of the ensemble. The percolation process is performed on a lattice formed by sites, a probability given as a parameter determines whether a site will become occupied and when two adjacent sites are occupied they become a cluster. These types of processes present a phase transition at a specific occupation probability, above which an infinite cluster is formed (over a theoretical infinite lattice). Below this critical probability only finite clusters are generated.

In the literature there are several applications of percolation to spatially constrained networks. Some efforts have focused on Erdös-Rényi networks^17, 18^; others use percolation as a means to investigate the robustness of the network^19^; and in some cases the emergence of regions^20, 21^. In addition, there already exists an approach to determine the hierarchy of main and secondary connections using percolation over the minimum spanning tree (MST) of a network^22^. This last approach performs well on the specific graphs studied in the paper (Erdös-Rényi, scale-free and grids), but it is not applicable to road networks since the main premise of the paper (that the MST contains the main roads) does not hold in road networks (the MST contains only parts of the main roads, leaving some or, depending on the case, even most segments of the highways out of the final graph). Within road networks specifically, percolation has been applied as an example to look at traffic behaviour^23^.

In previous work^8^, we considered the metric distance between intersections of roads as the threshold along which the percolation process occurred. In this work we propose a novel methodology, where the relative angle between road segments corresponds to the occupation probability in the percolation process and such percolation is performed on the graph of the road network (see Methods section) for more details on the transformations applied to the road network, in order for it to be used as our lattice). Relative angles between road segments have been part of the literature on road network analysis for a long time. These have been used to classify different cities’ typologies^24–26^, and also, they have been used to generate different representations of road networks. In ref. 27–29, the relative angle between segments is used to generate the dual informational graph which is then use to characterise the network behaviour. Moreover, a whole discipline, *Space Syntax*, has emerged from looking at road networks through centrality measures which are based on relative angles, and which serve to infer route choice, and urban structure^30–32^.

In the next section we show that we can identify different growth regimes, from which we can extract the main skeleton of the network, and devise a classification for each of the road segments. The methodology to obtain these results and the calculation of the critical exponents is described in the Methods section.

## Results

Our methodology shows that the system undergoes a continuous phase transition, and hence behaves in a critical way. This transition marks the point at which the giant cluster appears allowing us to extract the main skeleton of the underlying road network. In the Methods section, we show that the transition takes place at a critical probability *p*_*c*_ given at an angle of ≈45.76 degrees. Further details of the phenomenological and theoretical results are given also in such section, where we compute the critical exponents for the system, and introduce the corrections for the finite size effects.

Our approach shows that by applying Shannon’s entropy to the distribution of cluster sizes we can distinguish several growth regimes in the percolation process and that the phase transition can be used to determine the point at which to extract the main skeleton of the network. Moreover, we use the entropy measurements to construct a hierarchical index for each of the road segments which provides us with a methodology for the hierarchical classification of road networks which can be computed in *O*(*n*) (for a detailed explanation please refer to the section of the Supplementary Information S1, where the algorithm is described).

### Growth regimes emerging from the angular percolation of road networks

Different growth rates can be identified as the percolation threshold is increased. To determine the regimes that characterise the formation of the giant cluster, we analyse Shannon’s entropy of the distributions of the cluster sizes at the different thresholds

1 $${H}_{j}=-\sum _{\forall i}{p}_{i,j}\,\mathrm{log}({p}_{i,j}),$$

where *H*_*j*_ is the entropy at threshold *j*, ${p}_{i,j}=\frac{\sum {M}_{i,j}}{M}$ is the fraction of the total mass of clusters of state (mass) *i* at threshold *j* over the total mass of the system. Evidently, our phase space holds all the possible states (masses) that our system can take, including sites that do not belong to any cluster.

**Figure 1:**
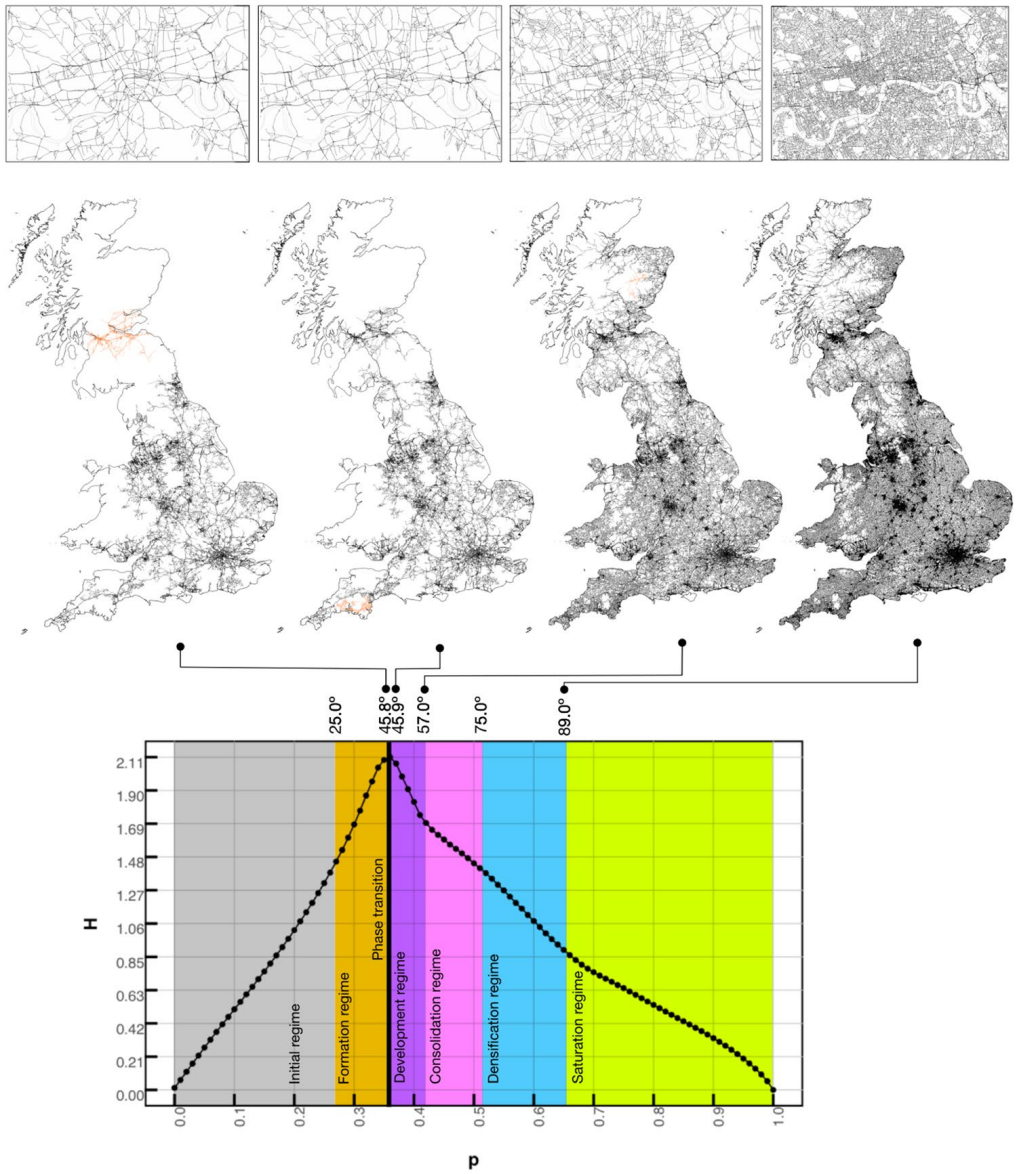
Shannon’s entropy of the distribution of the cluster sizes. We can clearly see 6 different regimes in the way the system behaves. Upper panel, zoom into the center of London. Center panel, giant cluster in black, second largest cluster in orange. Lower panel, entropy measurement and the different regimes.

Plotting *H*_*j*_ against the probabilities of each threshold *j* (Fig. 1), we observe that we can relate different speeds of variation in the entropy levels of the distribution of the clusters, to the different slopes of the curve. The change in slope determines the boundaries for the different regimes in terms of its percolation threshold. We identify 5 different regimes:

1. The initial regime in which the size of the giant cluster can be disregarded and only very small clusters are formed.
2. The formation regime, this corresponds to the formation and growth of the giant cluster, which will become infinite at the phase transition, and span over the whole system (from Scotland to the southern part of England). The maximum entropy is reached at the phase transition, by the end of this regime.
3. The development regime which starts at the phase transition at 45.763 degrees and ends approximately at 57 degrees. In this regime the giant cluster spans the whole UK incorporating the most important roads.
4. By the end of the consolidation regime all A and B roads with the most important minor roads (those with a dual carriageway) are incorporated to the giant cluster. This regime ends at approximately 75 degrees.
5. The next regime is the densification of the giant cluster, this marks the beginning of the incorporation of local roads to the network. Once this phase ends, at approx. 90 degrees, the most important local roads have also been included.
6. The last regime corresponds to the saturation regime, where the rest of the local roads and alley-ways are included into the giant cluster.

**Figure 2:**
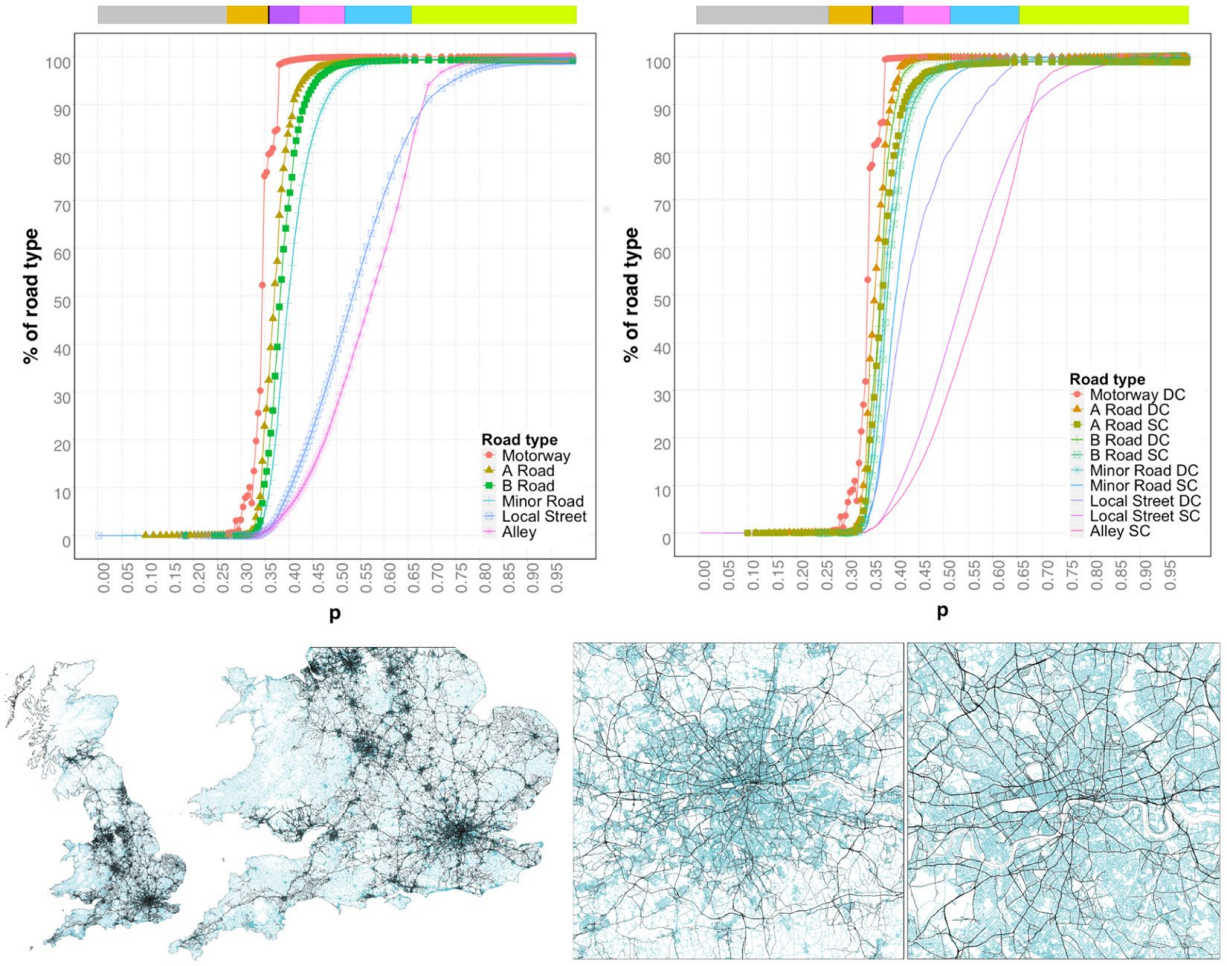
Upper panel, percentages of roads by road type that belong to the giant cluster at the different percolation thresholds (in right panel, DC: dual carriageway, and SC: single carriageway), the colors on top of the plots represent the regimes obtained with the entropy. Lower panel, giant cluster just after the phase transition (in black) overlaid to the full set of roads of the road network (in light blue).

The physical process taking place during the angular percolation is illustrated in Fig. 2. As the percolation threshold increases, the giant cluster sequentially incorporates the roads by importance. At the phase transition, the main skeleton of the road network appears. This contains the main important street segments: motorways, A, B and minor roads. After the transition, local roads and alleys get progressively incorporated to the giant cluster. The differentiation of importance of each street segment is further marked by the speed of integration into the giant cluster.

A very interesting observation is that when the roads are disaggregated by its secondary classification into dual/single carriageways (DC/SC), we can see (right panel of Fig. 2) that the different regimes identified using the entropy analysis actually correspond to different stages in terms of the types of roads that are incorporated to the giant cluster. At the phase transition the percolation incorporates to the giant cluster all the motorways. The end of the development regime corresponds roughly to the moment where not only the motorways but also A roads (DC) and B roads (DC) are completely incorporated into the giant cluster. The end of the consolidation regime corresponds to the point where simultaneously the A roads (SC), B roads (SC), and the most important minor roads (DC) are incorporated into the cluster. By the end of the densification regime also the rest of the minor roads (SC) and the most important local roads (DC) belong to the giant cluster. The end of the saturation regime corresponds to the inclusion of the rest of the roads (local roads (SC) and alleys) into the giant cluster.

### Identifying the skeleton of the network

The roads that are in the giant cluster just after the phase transition (which happens at 45.763 degrees, see Table 1 of the Methods section) are portrayed in black in the lower panel of Fig. 2, while the full set of roads are in light blue. It is easy to see that the giant cluster after the phase transition contains the main roads of the system.

**Table 1:** Critical exponents obtained for the UK road network: before (Finite system), and after introducing corrections (Infinite system) due to the finite size of the system.

Exponent	Finite system	Infinite system
*β*	0.6186(*)	0.6187(*)
*γ*	2.8100	2.9053(*)
*ν*	2.2182	2.2706(*)
*τ*	2.1804	2.1763
*σ*	0.2917	0.2849
*D*	1.5456(*)	1.5456
*d*	1.8245(*)	1.8245
*p*_*c*_	0.3783(*) [45.763^*o*^]	0.3766(*) [45.39^*o*^]

The values marked with (*) are the ones that have been calculated from the experimental results, the rest of the exponents were calculated by using the scaling relationships between the exponents.

Let us quantify this by looking at the percentages of roads included. Just after the phase transition, despite the fact that the giant cluster has a mass of only 17% of the full road network, it already contains 98.3% of the Motorways, 66.9% of the A roads, 47.7% of the B roads, 28.8% of the minor roads while only containing 0.50% of the local roads and 0.35% of the alleys. This shows that after the phase transition, the giant cluster corresponds to the skeleton of the road network, containing the major roads.

Furthermore, if we disaggregate the roads by their secondary classification (single or dual carriageway, SC/DC) we observe that after the phase transition the giant cluster contains 99.5% of the Motorways (DC), 81.5% of A roads(DC), 71% of B roads (DC) and 52.4% of the minor roads (DC). Our approach creates a hierarchical division of the roads, from which we can derive in a natural way the skeleton of the network without having to use the road classification. The skeleton is obtained by extracting the giant cluster at the consolidation phase, as close as possible to the critical probability in order to include the minimum number of roads.

### Hierarchical index for road segments

The hierarchical classification of road networks is of fundamental importance to establish the routes with the highest probability to render a fast connection between nodes. Moreover, this is used for navigational purposes, to aid drivers in the identification of the most probable routes between destinations^33^. For this reason, several algorithms for the detection of shortest routes in road networks have been developed, and are highly dependent on the classification and the hierarchical organisation of the road networks^34^. On top of this, when comparing road networks from different countries we are bound to find different classification systems rendering the quest to establish an equivalence between them extremely difficult. There is, therefore, a large interest in the generation of a methodology that automates the hierarchical classification of road networks.

Centrality measures have played a major role in the description of systems of road networks allowing to uncover a large number of its properties. As such, one of the most accepted methodologies to visualize the hierarchical organisation of the road network is the use of betweenness centrality^35^. This gives a value for each road proportional to its flow through the system. A large body of research has been devoted to the analysis of flows using centrality measures^36, 37^, and, in the case of road networks, a large part of that research has been devoted to the use of angular distances^31, 38^, which seems to improve the detection of these main flows^39^. The large drawback of using betweenness to generate an index is that the complexity of its computation (≈*O*(*n* ^3^), where *n* is the number of nodes of the graph) makes infeasible its use for large systems. Another methodology that we can find to determine the hierarchy of reticular networks using a graph theoretic approach is the one found in ref. 40 that studies its application in biological networks.

In this section we propose an alternative methodology to generate a hierarchical index for each road segment that is based on the percolation process. This technique is computationally less demanding than calculating the betweenness index, and it is linear in complexity (≈*O*(*n*)) which means that it is fully capable of analysing large systems. We construct the index according to the following principle. This consists in assigning a degree of importance to the street segment according to its contribution to the informational content of the system. To do this, we weight the entropy of the system with the mass of the cluster to which the street segment belongs. Its total contribution needs to be considered for each percolation threshold, hence we sum over all the thresholds (for a discussion on how to obtain the best possible unbiased set of thresholds, please refer to the Supplementary Information S2.1). Given that the cluster sizes follow a power law distribution (this behaviour is further studied in the section of the Supplementary Information S2.2), it is more appropriate to weight the entropy with the log of the mass instead of the mass itself. More formally we can write that the normalised hierarchical index *I*_*i*_ of road *i* is:

2 $${I}_{i}=\frac{\sum _{j\mathrm{=1}}^{t}{H}_{j}\,\mathrm{log}({M}_{i,j})}{\sum _{j\mathrm{=1}}^{t}{H}_{j}log(max({M}_{j}))}$$

where *j* runs through all the percolation thresholds (*t*), *M*_*i*,*j*_ is the mass of the cluster that contains road *i* at the *j*-*th* threshold, *H*_*j*_ is the entropy of the distribution of the cluster sizes at the *j*-*th* threshold and *max*(*M*_*j*_) is the maximum mass of all clusters at threshold *j*.

**Figure 3:**
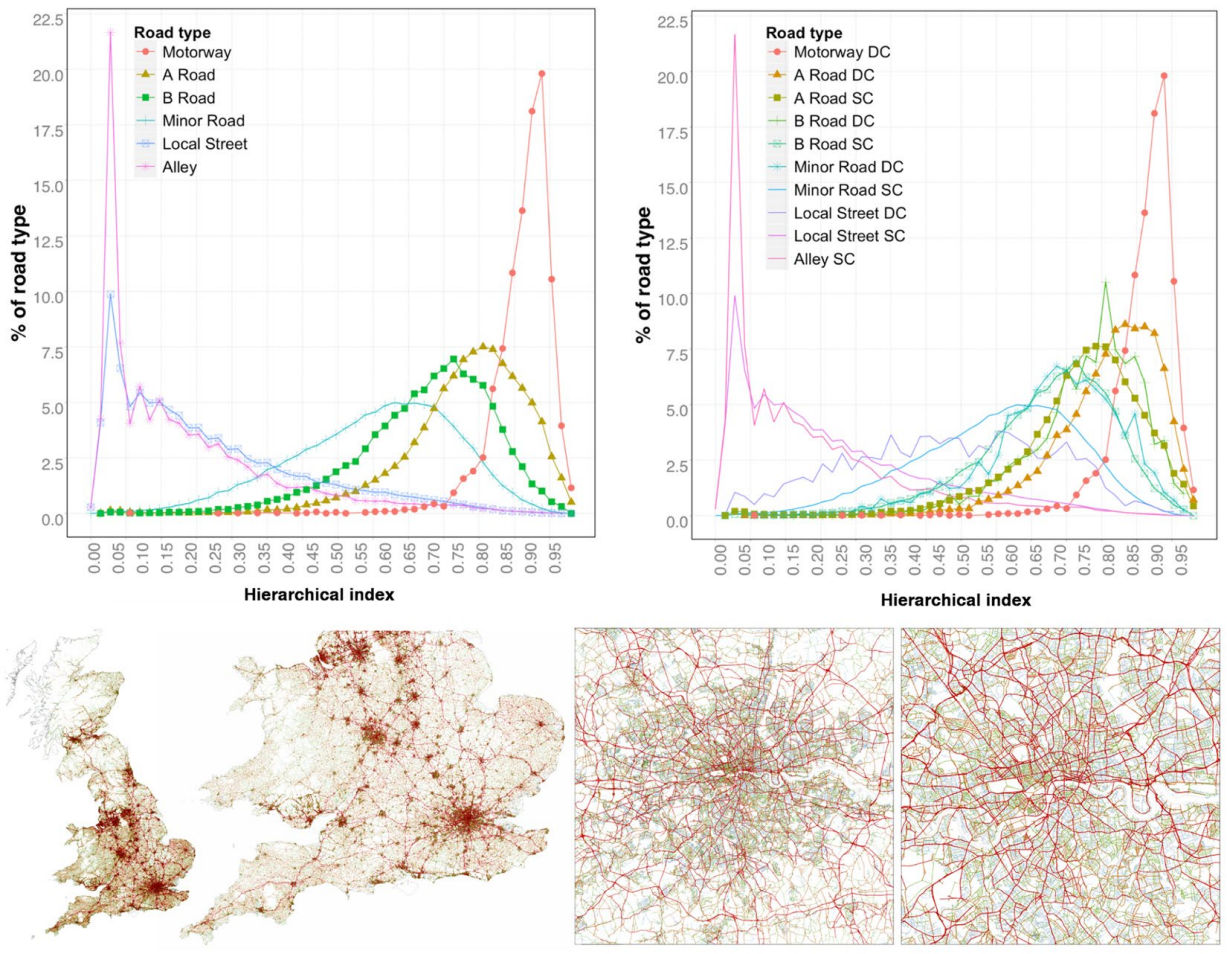
UK’s road network at different scales where the thickness of the lines and the color scale correspond to the values of the hierarchical index as calculated using Equation (2).

The results of our index are shown in Fig. 3. We can see that roads have been assigned an index that is consistent with the given road classifications (the highest values correspond to Motorways, then A-roads, followed by B-roads, minor roads, local streets and alleys). For further granularity, we look at the sub-classifications differentiating between DC and SC. Observing the right upper panel, we can see that the index classifies with the same histogram A-roads (SC) and B-roads (DC), and the same holds true for B-roads (SC) and minor roads (DC). Meanwhile, A-roads (DC) get closer to their classification as Motorways. These observations hint that though their classification is a priori different, the fact that internally (in the comparison between the secondary classification SC/DC) dual-carriageway roads are more important than single ones affects their hierarchical position within the system.

## Discussion

Throughout this work we have shown that the road network viewed as a percolation process behaves in a critical way. The phase transition marks a differentiated growth rate, and the threshold at which the giant component can be identified with the skeleton of the road network. Furthermore, we observed a hierarchical organisation on the importance of the road segments with respect to their contribution to the entropy of the system at each threshold of the percolation. This was used to construct a hierarchical index for each road segment that serves to classify the different roads in the network.

In conclusion, we have shown that the road network encounters a natural description when seen as a percolation process, where the threshold corresponds to the angle between the street segments. These are very promising results that open up new possibilities for further studying the road network properties under this paradigm. Furthermore, the hierarchical index can serve as the basis for an algorithm in the spirit of contraction hierarchies that could improve the speed of shortest path analyses. These are properties that cannot be observed in the system under its metric description and that, therefore, highlights the angular nature of the road network.

A description of the percolation process in terms of the critical exponents has also been studied, including the corrections for the exponents taking into account the finite size effects. In future work, we intend to apply this process to other systems, such as road networks from different continents and natural systems, in order to establish a basis for comparison through the critical exponents of the infinite lattice. This will give us insight into whether these systems share generic properties, or whether a classification can be achieved; in addition to investigate whether growth processes can be inferred from these behaviours.

## Methods

The system under consideration is the UK road network. The original data is obtained from the OS MasterMap database (ITN layer)^41^. The layer is processed and simplified as follows: roundabouts are collapsed into a single node, lanes are collapsed into a single link without considering directionality, and the nodes of degree 2, which correspond to intermediate points are removed. Only nodes corresponding to intersecting roads are kept. The simplified version of the network can be found in the data repository^42^. Note that by removing these nodes more emphasis is put on the structural properties of the network, since the angles within a road are not considered.

The properties of road networks can be divided into two subclasses: structural and geometrical. The former are studied by representing the network as a graph, where the nodes are the road intersections and the links are the street segments. This allows us to calculate many network features: e.g. the degree distribution, centrality measures, the spectrum of the graph, or to extract communities, among many. Geometrical properties, in turn, are related to distances over the network, to lengths of the street segments, the width of roads, the slopes formed by the topography, the relative angle between two street segments, and so forth. One of the most common ways to incorporate these geometric factors into the analysis is to include them as weights in the graph of the road network.

Note that these weights refer to a relationship between the nodes in the graph, as is the case of the length of roads, but in our case, the relative angle is a relationship between the links of the graph (the road segments). In order to account for this second type of relationship, we need to generate the link-node dual (or line-graph^43^) of the road network’s graph.

**Figure 4:**
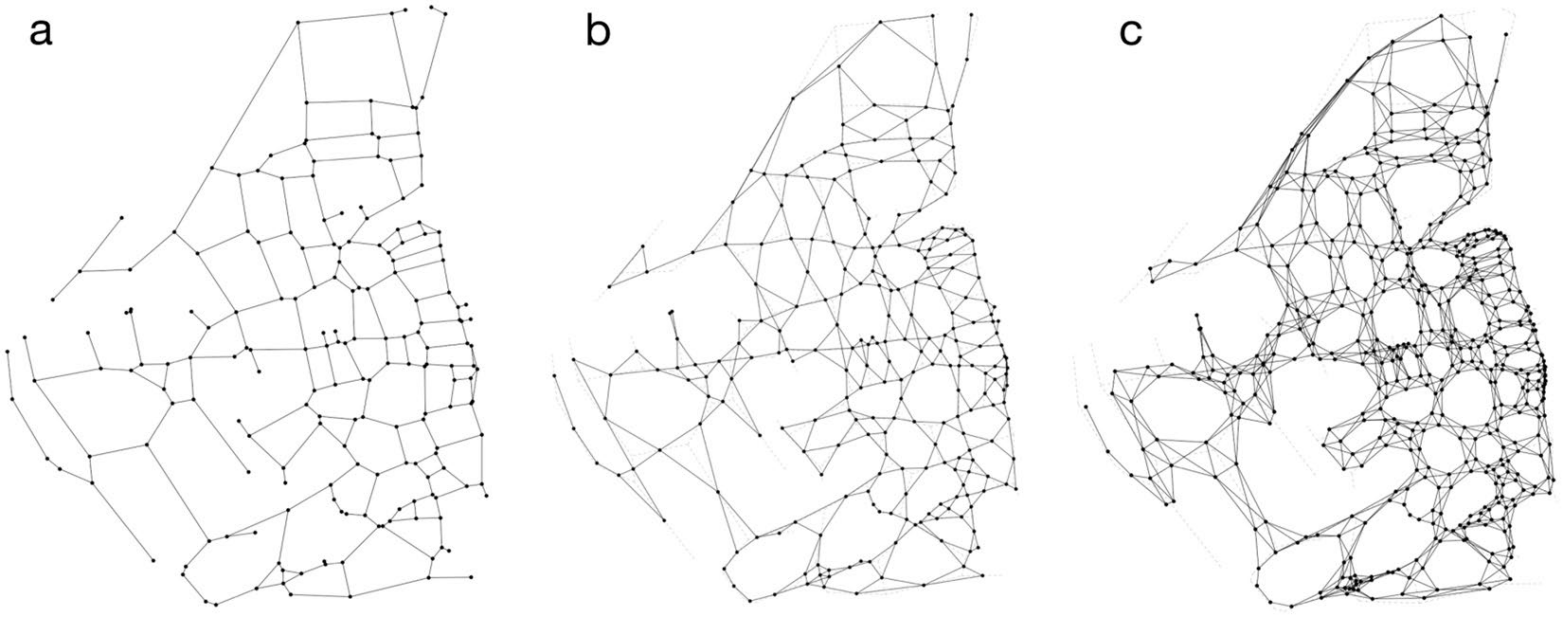
(**a**) Example of a primal graph of a street network (*G*), (**b**) line-graph generated from that primal graph (*G*′ = *L*(*G*)), (**c**) line-graph of the line-graph (*G*″ = *L*(*L*(*G*))) which is the lattice of our percolation.

Let us denote by *G* the graph of a road network (Fig. 4a), its line-graph, denoted by *G*′ = *L*(*G*), is constructed as follows (Fig. 4b). Each link (street segment) in the original graph, is replaced by a node in the line-graph, and a link is created in the line-graph if two links of *G* share a node. The line-graph generated from the network holds 4 million nodes and 7 million links after processed and simplified. The relative angle between road segments can now be mapped to the weights of the line-graph. This procedure is very similar to the one presented in ref. 31, although here we do not normalize the values of the relative angles.

**Figure 5:**
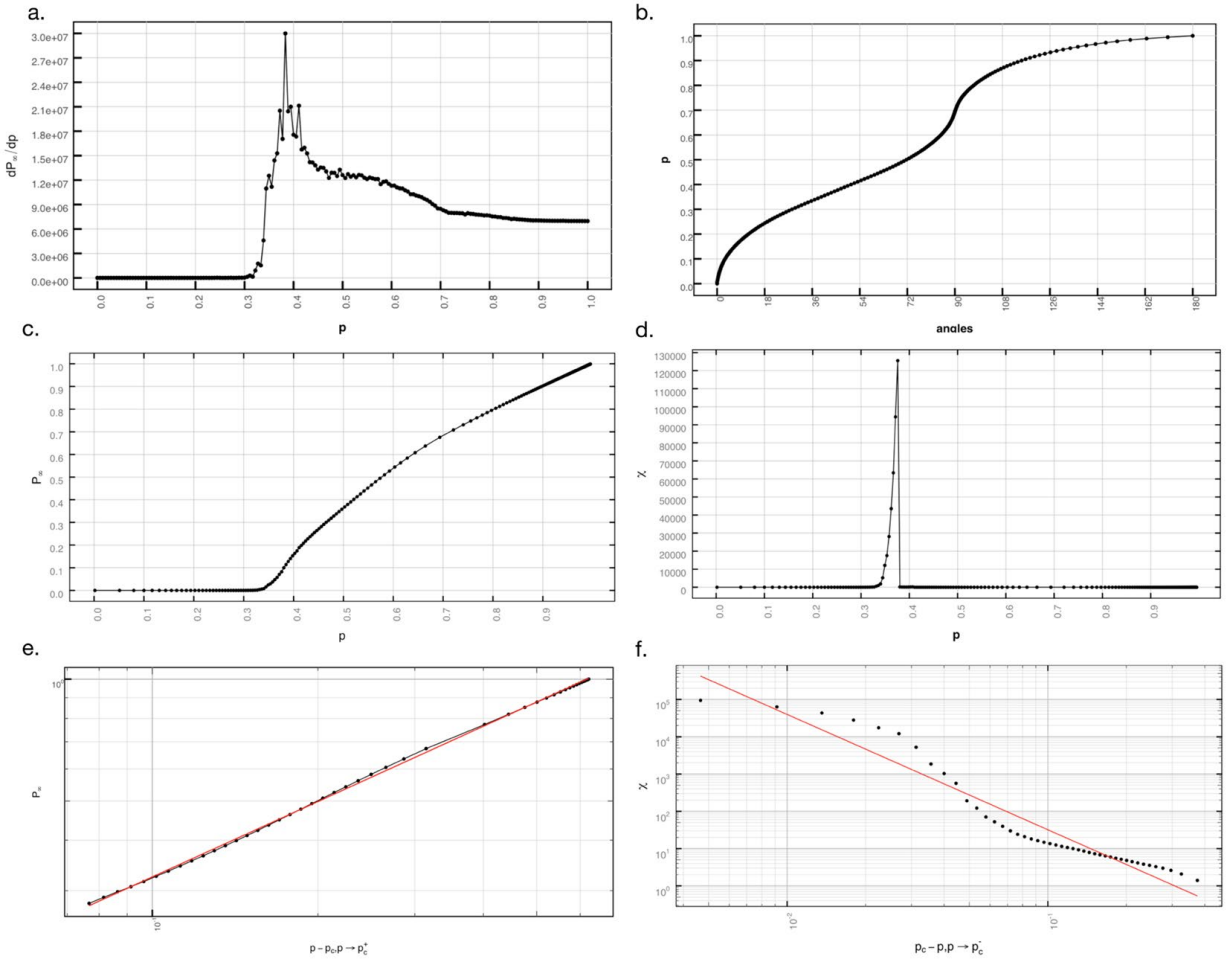
Critical behaviour of the UK road network: (**a**) derivative of the order parameter; (**b**) relationship between each angle and its cumulative probability of occurrence; (**c**) order parameter *P*_∞_; (**d**) average cluster size *χ*; (**e**) critical exponent *β*; and (**f**) critical exponent *γ*.

The bond percolation is then executed on the line-graph (*G*′) of the street network as follows. Given a certain angular threshold, the occupation probability for a link is the probability that a link of the line-graph has a weight equal or below the angular threshold (Fig. 5b). We can then associate a probability to the angular threshold by computing the percentage of links that are below or equal to this threshold. For example, the probability associated to 45 degrees is *p* = 0.376 since 37.6% of the links are below or equal to 45 degrees (see Figure S1b).

Taking into account that what we are calculating is a bond percolation on the line-graph, we can consider that conceptually we are performing a site percolation on the line-graph of the line-graph (Fig. 4c) where every node will correspond to a site of the lattice used for the percolation, and the probabilities of each site to be occupied are proportional to the weights of the line-graph. This *L*(*L*(*G*)) will serve us as the equivalent of the lattice in a typical percolation process and we will use it to calculate the fractal dimension of our system. Throughout the text we will refer to the number of links in the line graph as the mass of the system (or say of a particular cluster), which is equivalent to the number of nodes of the *L*(*L*(*G*)).

The interested reader can find all the specificities of the algorithm to calculate the percolation process in the section of the Supplementary Information S2.

### Percolation theory and phenomenology

Percolation processes are critical phenomena that present continuous phase transitions that can be characterised by critical exponents^44^. In this section we show how these exponents can be described and computed for our system. We correct for any errors arising from finite size effects in our calculations through the introduction of a series of subsystems of different sizes.

### Critical exponents

Critical phenomena have been largely studied in physical systems, in particular in systems where the temperature *T* defines the various phases, and the critical point at which the transitions take place^45^. For these systems in general, a full ordered system can be found at *T* = 0, and as this is increased, it reaches a critical point *T*_*c*_ at which the system undergoes a continuous phase transition, and the correlation length diverges *ξ* → ∞. As the temperature is increased, at the asymptotic limit *T* → ∞ the system is fully disordered. In percolating systems, the occupation probability takes the role of the temperature, and at a critical probability *p* = *p*_*c*_, the system undergoes a second order phase transition and a giant cluster appears^46^ spanning the whole space. At this point the percolation clusters become self-similar, and the system can be described as a fractal^47^. This transition can be fully characterised in terms of critical exponents which only depend on the dimension of the space and type of percolation^46^. Although several exponents can be defined, only two of them are independent, and the rest can be obtained through a series of scaling laws.

Cities are known to have a fractal structure as demonstrated a few decades ago by^48, 49^, and more recently they have been considered as multifractals^50, 51^. We will hence consider that our system (the road network) is a fractal and define the fractal dimension *d* of our system as the capacity dimension of the *L*(*L*(*G*)) of the road network graph. This fractal dimension will be measured by using the box-counting methodology on the nodes of the graph *L*(*L*(*G*)). This allows us to establish the relationship *M* = *l* ^*d*^, where *M* corresponds to the mass of the system, in this case the number of nodes of *L*(*L*(*G*)) (equal to the number of links of the *L*(*G*)), and *l* to the theoretical lattice length. This equivalence allows us to calculate a theoretical lattice size (which will be needed to remove finite size effects later on)

3 $$l={M}^{\frac{1}{d}}$$

At the percolation threshold *p*_*c*_, the giant component spans the whole system and becomes self-similar^44, 46, 47^. The fractal dimension *D* of the largest component at the percolation threshold can be obtained in a similar way

4 $${M}_{\infty }({p}_{c},l)={l}^{D}$$

where *M*_∞_(*p*_*c*_, *l*) is the mass of the spanning cluster at the phase transition, and *l* is the size of the lattice. Moreover, since ${P}_{\infty }=\frac{{M}_{\infty }}{M}$ we can say ${P}_{\infty }({p}_{c},l)=\frac{{l}^{D}}{{l}^{d}}$ or *P*_∞_(*p*_*c*_, *l*) = *l* ^*D−d*^. Equation (4) can be used to calculate *D* given that we can directly measure *M*_∞_ and we can calculate *l* (equation (3)) then $D=\frac{log({M}_{\infty })}{log(l)}$.

In a typical percolation process, the probability of a site belonging to the giant cluster ${P}_{\infty }=\frac{{M}_{\infty }}{M}$ (Fig. 5c) takes the role of the order parameter. It is practically 0 below the phase transition and increases rapidly after, reaching a fully ordered system at *p* = 1.

This quantity will allow us to calculate the critical probability *p*_*c*_ at which the system undergoes a phase transition. In order to find the location of the phase transition we detect the maximum of the derivative of our order parameter as shown in Fig. 5a (another methodology to calculate *p*_*c*_ would be to look for the threshold that maximises the mass of the second largest cluster). For the UK road network we obtain *p*_*c*_ at an angle of 45.7631 ± 0.0001 degrees, which corresponds to the probability *p*_*c*_ = 0.3783.

The behaviour of *P*_∞_ at the critical point is characterised by the critical exponent *β* (Fig. 5e) as follows

5 $${P}_{\infty }\propto {|p-{p}_{c}|}^{\beta },p\to {p}_{c}^{+}$$

The clusters that appear at the different probabilities *p* < *p*_*c*_ are characterised by their linear dimension *ξ* ^52^. This is one of the most important variables in critical phenomena, containing the information of the range of correlations given by the interactions. At the phase transition, the correlation length diverges, *ξ* → ∞. Its behaviour close to the critical point is also a power law leading to the critical exponent *ν*

6 $$\xi \propto {|p-{p}_{c}|}^{-\nu },p\to {p}_{c}$$

Let us look at other important quantities in percolating processes that give rise to these exponents. The clusters that appear at the different probability thresholds can be characterised according to their average size *χ*. This is defined as follows

7 $$\chi =\frac{\sum {s}^{2}{n}_{s}}{\sum s{n}_{s}}$$

where *s* is the cluster size and *n*_*s*_ the normalised number of clusters of size *s* per site (the number of clusters of size *s* normalised by the total number of sites in the lattice). The average cluster size increases as *p* increases, until the critical probability *p*_*c*_ is reached. Once this happens, the giant clusters spans the system, and the average cluster size drops suddenly, since above the critical probability the giant cluster is infinite and therefore is not taken into account, see Fig. 5d. The critical exponent *γ* associated with *χ* hence measures the increase and decay of the size of the finite clusters around the critical probability *p*_*c*_, and this is obtained from the following equation

8 $$\chi \propto {|{p}_{c}-p|}^{-\gamma },p\to {p}_{c}$$

see Fig. 5f. The typical size of the largest cluster *s*_*ξ*_ is referred as the characteristic cluster size, and it can be obtained in a similar way

9 $${s}_{\xi }\propto {|{p}_{c}-p|}^{-\frac{1}{\sigma }},p\to {p}_{c}$$

where the exponent *σ* determines the speed of the variation of the characteristic cluster size. Lastly, the Fisher exponent *τ* characterizes the variation of the normalized number of clusters around *p*_*c*_.

10 $${n}_{s}\propto {s}^{-\tau }\cdot {\mathscr{G}}(s/{s}_{\xi })$$

where *s* is the size of the cluster and *s*_*ξ*_ is the characteristic cluster size and ${\mathscr{G}}(s/{s}_{\xi })$ is the scaling function for the cluster number density and is defined as ${\mathscr{G}}(s/{s}_{\xi })={(s/{s}_{\xi })}^{2}\cdot exp(-s/{s}_{\xi })$.

These exponents are related to each other according to some scaling laws, and from all this collection, only two are independent. We can therefore use these scaling laws to analytically calculate the rest of the exponents once we have measured two of them. The scaling laws derived from taking *D* and *β* as the independent exponents are:

11 $$\nu =\frac{\beta }{d-D}$$

12 $$\gamma =\nu \mathrm{(2}D-d)$$

which lead to the well-known variation of Rushbrooke inequality^52^

13 $$2\beta +\gamma =\nu d$$

In terms of *σ* and the Fisher exponent (*τ*), we have

14 $$\sigma =\frac{1}{\nu D}$$

15 $$\tau =\beta \sigma +2$$

The initial results are given in Table 1 under the column “Finite system”. These results do not take into account corrections that need to be introduced given the finite size of the system.

In the following section we introduce these.

### Removing the finite size effect

Correcting for finite size effects is not a trivial matter. One methodology that can be employed^53^ is to consider different sizes of the system and perform the analysis considering that the results are valid for the asymptotic limit, which in this case corresponds to having the size of the system much larger than the correlation length. Effectively we are re-scaling the results, so we obtain a data collapse.

We generate 32 subsets of different size of road networks from the original one. Each of those systems will have a different mass and lattice size, but will maintain the same fractal dimension. Following Equations (5) and (6), we can write *P*_∞_ as a function of the correlation length at the asymptotic limit

16 $${P}_{\infty }\propto {\xi }^{-\frac{\beta }{\nu }},p\to {p}_{c}^{+}$$

**Figure 6:**
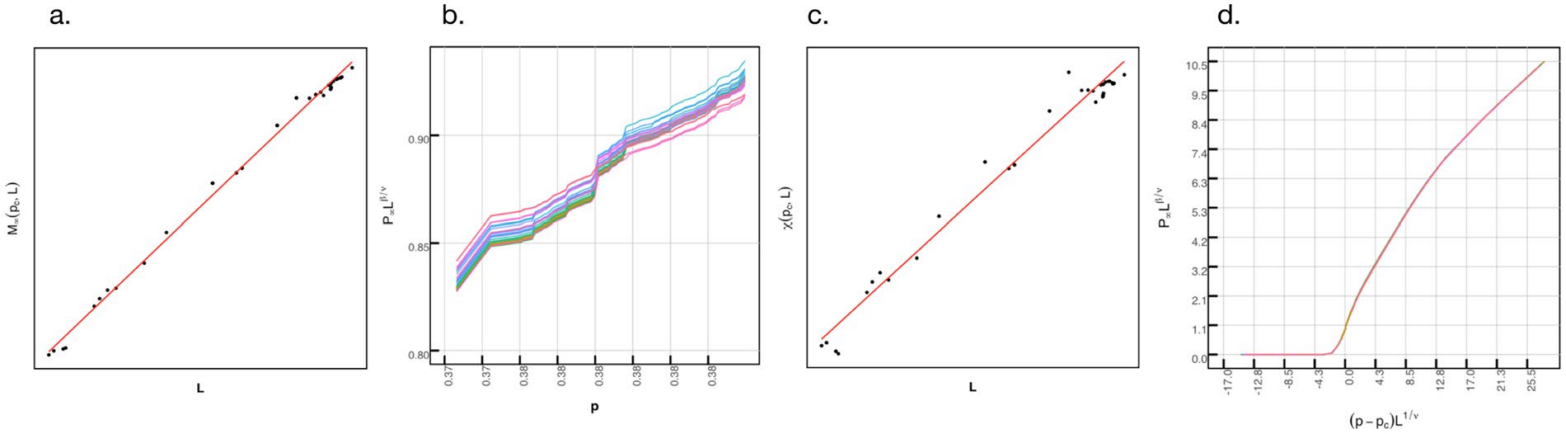
Calculations correcting for the finite size effect (infinite theoretical system), from left to right, (**a**) calculation of *D*, (**b**) correction of $\frac{\beta }{\nu }$ and simultaneous determination of *p*_*c*_, (**c**) calculation of $\frac{\gamma }{\nu }$, (**d**) data collapse to calculate *ν*.

At *p*_*c*_ the correlation length *ξ* becomes larger than any of the lattice sizes of our finite systems, and the equation no longer holds. We solve this by capping the size of the largest cluster at *p*_*c*_ by the lattice size as follows: ${P}_{\infty }({p}_{c},l)\propto {l}^{-\frac{\beta }{\nu }}$, which is equivalent to: *P*_∞_(*p*_*c*_, *l*) ∝ *l* ^*D*−*d*^. Therefore, we can obtain an initial estimate for $\frac{\beta }{\nu }$ by taking a measure of the slope of a log-log plot of *M*_∞_ against *l* for all the subsystems (Fig. 6a) which gives us an estimate of *D* (equation (4), that can then be inserted into $-\frac{\beta }{\nu }=D-d$.

We should remark that the critical probability in an infinite lattice will probably be different from the previously calculated one in our finite system. In order to find *p*_*c*_ for the infinite case, we use the methodology proposed in ref. 54. This consists in plotting ${P}_{\infty }({p}_{c},l)\cdot {l}^{\frac{\beta }{\nu }}$ vs. *p*, and adjusting the value of $\frac{\beta }{\nu }$ until all the curves cross in one single point (Fig. 5b). That point will be our *p*_*c*_ and the final value of $\frac{\beta }{\nu }$ will be given by this procedure.

In a similar manner, using Equations (6) and (8), and capping the size of the largest cluster at *p*_*c*_, we can calculate *χ*(*p*_*c*_, *l*) at different lattice sizes at the critical probability so that

17 $$\chi ({p}_{c},l)\propto {l}^{\frac{\gamma }{\nu }}$$

allowing us to determine $\frac{\gamma }{\nu }$, see Fig. 6c).

Let us now determine *ν* correcting for the finite size effect. For all the different lattices, we plot ${P}_{\infty }\cdot {l}^{\frac{\beta }{\nu }}$ vs. $(p-{p}_{c})\cdot {l}^{\frac{1}{\nu }}$ (Fig. 6d) and adjust the value of $\frac{1}{\nu }$ until all the data collapses. This gives us the value of *ν* and with the calculated values of $\frac{\beta }{\nu }$ and $\frac{\gamma }{\nu }$ we can obtain *β* and *γ*. The fractal dimension *D* can be computed using Equation 12 The scaling laws (14), (15) can be used to find *σ* and *τ*. The results for the “Infinite system” can be found in Table 1.

### Software

The Figures in the paper were generated using the following open-source software platforms and libraries:

- ggplot2 2.1.0^55^ (Figures 1–3, 5 and 6)
- R 3.3.3^56^ (Figures 1–6),
- RStudio 0.99.903^57^ (Figures 1–6),
- QGIS 2.4^58^ (Figures 1–3)
- Inkscape 0.91^59^ (Figures 1–6).

## Electronic supplementary material

SupplementaryInfoAngularNatureRoadNetworks
