# Supplementary material for: The angular nature of road networks
Source: Sci Rep. 2017 Jun 27;7:4312. doi: 10.1038/s41598-017-04477-z (PMC5487334; doi:10.1038/s41598-017-04477-z)
Supplement: Supplementary file 1 — SupplementaryInfoAngularNatureRoadNetworks [file 41598_2017_4477_MOESM1_ESM.pdf]

The angular nature of road networks: supplementary information

Carlos Molinero[1,*], Roberto Murcio [2], and Elsa Arcaute [1]

[1]Centre for Advanced Spatial Analysis (CASA), UCL
[2]Consumer Data Research Centre (CDRC), UCL
[*]c.molinero@ucl.ac.uk

ABSTRACT

This section contains the algorithm used to compute the percolation process using the relative angle between street segments as the occupation probability, and the method to obtain the right thresholds in order to compute the hierarchical index.

S.1 Algorithm for the angular percolation on the road network

Given a graph of the road network $G = (V, E)$, where V represents the set of intersections and E represents the set of streets, we generate its line-graph and assign the angular distance between streets as the weights of the links of the line-graph w ($G' = (V', E', w)$). For a certain angular threshold (t) (e.g. $t = 40$ degrees) we produce a network percolation as follows:

1. Delete all links that are above the threshold.

2. Find the connected components of the resulting graph. Each of those components will be a cluster of the percolation.

This simple algorithm is $O(n)$, given the nature of the system. Road networks' graphs have very specific characteristics, they are planar, sparse and due to spatial restrictions (i.e. the width of the streets) their maximum degree is very limited (to around $k_{max} = 10$). This gives the following results:

- The number of links in a planar graph is linear with respect to the number of nodes $m = c \cdot n$ where m is the number of links, n the number of nodes and c a constant.

- The complexity of generating a line graph is determined through three steps. First, all links need to be considered in order to generate the nodes; secondly, all nodes need to be considered in order to generate the new links; and finally, all the links that depart from the end nodes need to be considered and their angles in order to assign the weights. In simple terms, we can say that the complexity has an upper bound of $\Omega(m + 2mk_{max})$, where k_{max} refers to the maximum degree of a node. This is linear in road networks since in these graphs the maximum degree can be considered constant $k_{max} = c$, so its complexity is more or less $O(m \cdot c)$; and because the graph is sparse the complexity is more or less linear in the number of nodes $O(n)$.

- The algorithm that generates the clusters following the percolation procedure is linear in time. The first step is linear in the number of edges and the second step can be implemented with a BFS (breadth-first search) which runs also in linear time ($O(n + m)$). The execution time will be of $O(m + n + m)$ which can be reduced to $O(n)$ for sparse graphs such as road networks. So the total execution time of the algorithm (including generating the line graph) in road networks is $O(n)$.

The algorithm will be executed t times, where t is the number of thresholds, which is independent of the input (n). Therefore, the complexity for executing the algorithm remains linear ($O(n)$).

S.2 Appendix to the calculation of the hierarchical index

S.2.1 Determining the set of thresholds

In this section we will explain how to determine the correct set of thresholds in order to compute the hierarchical index. As we can intuitively understand from its formulation, the hierarchical index will give different results depending on the set of thresholds chosen for its calculation. We will try to generate a set of thresholds that gives us maximum information, with the

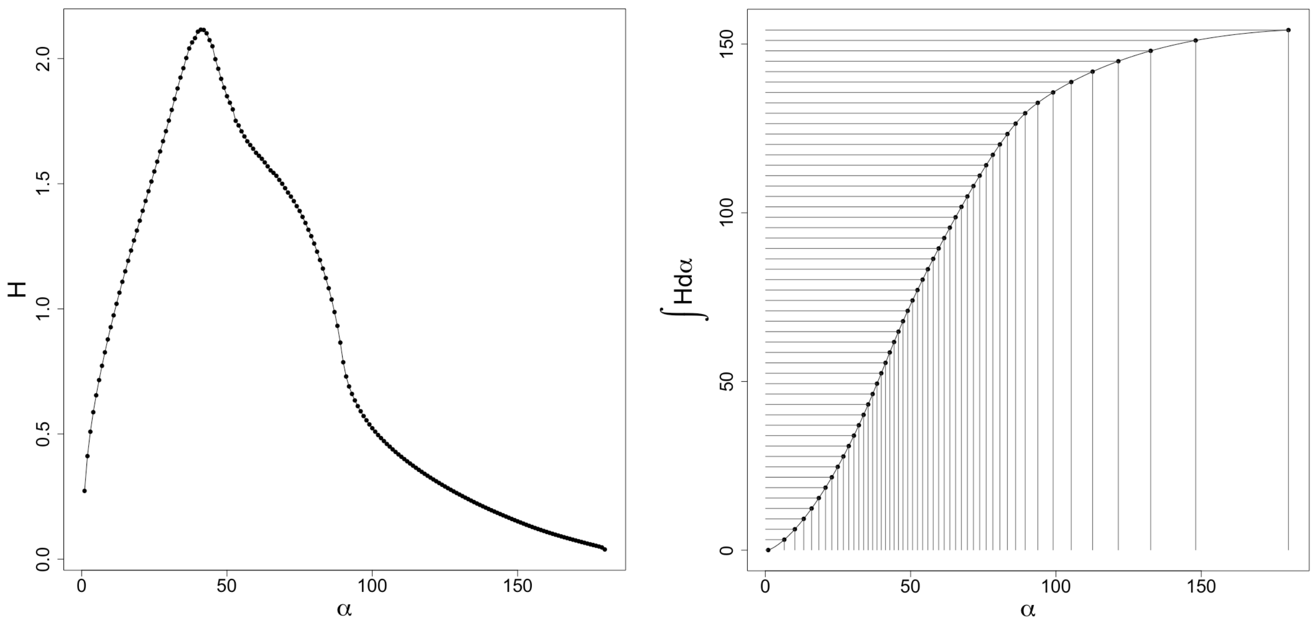

Figure S1. Left, entropy of the sizes of the percolation against the angle used as a threshold. Right, Obtaining the set of thresholds by dividing in equal parts the integral of the entropy. In this figure α is the angle that acts as a threshold of the percolation process.

least number of computations and an even spread of the thresholds over the set of possible values. An initial naive approach would be to just generate a set of thresholds such that every angle is a threshold, but this will have two small setbacks: on the one hand we will be calculating thresholds that do not contribute much to the calculation since their entropy is too low; and on the other, using an even distribution of the thresholds given by the angles does not guarantee a correct subdivision of the space. In this spirit, we can use the entropy to derive a set of thresholds that partitions the space such that their contributions towards the hierarchical index are meaningful, and small variations can cause a large modification depending on the value of the entropy, say lower when the angle is close to 0 or 180 degrees.

To obtain this set of thresholds we calculate the integral of the entropy for every angle (see Fig. S1) and then subdivide in equal parts the y axis (which will depend on the desired definition, in our case we used 100 parts). This returns the desired set of thresholds in the x axis (see vertical lines in the right panel of Fig. S1).

The reasoning behind this method is that the location of the breaks will depend on the slope of the curve (breaks will concentrate where the slope is higher), which is the derivative of our function which in turn is our entropy. Therefore, this procedure will generate a larger number of subdivisions where the entropy is higher and less where it is lower but will still span over the whole set of thresholds. To get an intuitive feeling about how this works, just imagine using the lowest possible definition (dividing the space into 2 parts), if we were to use the angles and divide them evenly we will get a set of thresholds [0,90,180] which carries very little information (remember that the phase transition is at 45.76), using this method the set of angles returned will be [0,52,180] and we can see that the newly included threshold (52 degrees) is closer to the phase transition so its entropy is higher, which will give a better approximation of the result of the hierarchical index when using a large number of thresholds. Of course as the definition (number of breaks) increases, the set of threshold becomes more correct but using this way of partitioning the thresholds we get a consistent result regardless of the definition used for the calculation.

S.2.2 Studying the power-law distribution of cluster sizes

As mentioned in the main text, the computation of the hierarchical index makes use of the logarithm of the cluster sizes instead of the sizes themselves, because the distribution of the cluster sizes follows a power-law. Let us employ the methodology developed in[1] in order to determine whether the distribution of cluster sizes at a certain threshold follows a power-law or not. In Fig. S2, we present the results of the analysis for 4 thresholds corresponding to 4 scenarios: 1) an example of an angle well below the phase transition (say at 25.00 degrees); 2) the angle corresponding to the maximum of the entropy (41.86 degrees); 3) at the phase transition (45.76 degrees); and 4) an angle above the phase transition (say 60.00 degrees). Following the results in[1], we observe that only for the latter case we can reject the hypothesis that the distribution follows a power-law

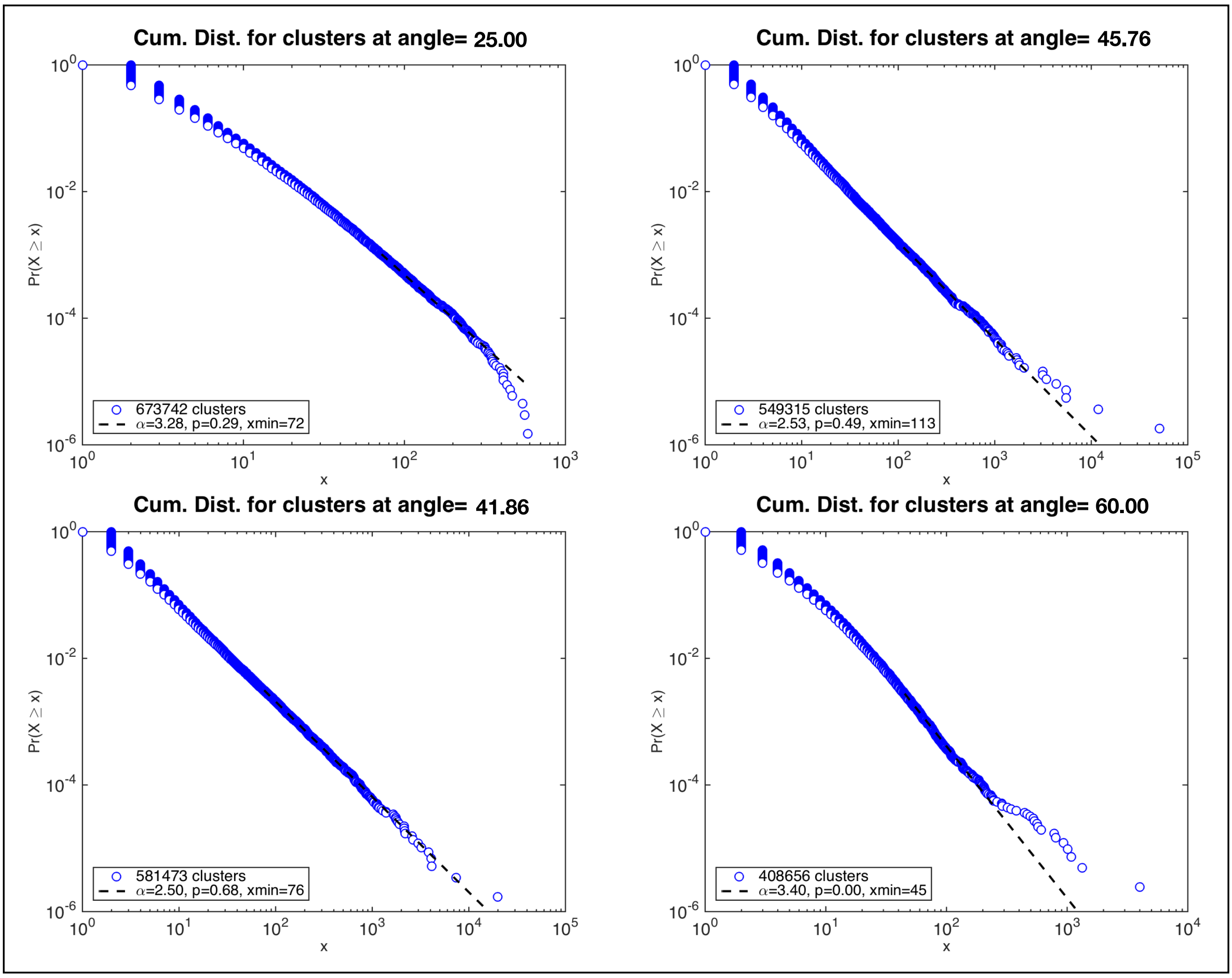

Figure S2. Cumulative distribution of cluster sizes at 4 different angular thresholds. Only for angle=60.00 degrees the hypothesis is rejected ($p \leq 0.1$).

(according to the goodness-of-fit test, if $p \leq 0.1$ the distribution cannot be modelled as a power-law). That is, well-above the phase transition, the cluster sizes are not distributed according to a power-law, nevertheless, it is obvious from the plot of the cumulative, that it is still necessary to transform the cluster sizes into their logarithm for the computation of the hierarchical index, so that larger clusters do not overshadow the computation, undermining the range of values.

References

1. Clauset, A., Shalizi, C. R. & Newman, M. Power-law distributions in empirical data. *SIAM Review* 661–703 (2009).
